# Robot-Assisted Simple Prostatectomy Versus Endoscopic Enucleation for Large-Volume Benign Prostatic Hyperplasia: A Systematic Review and Meta-Analysis of Perioperative Outcomes and Complications

**DOI:** 10.3390/jcm15135276

**Published:** 2026-07-06

**Authors:** Sophia Tsokkou, Manuela Sieberer, Ioannis Konstantinidis, David Oswald, Antonios Keramas, Christian Ramesmayer, Julia Katharina Peters, Lukas Lusuardi, Chrysovalantis Toutziaris, Petros Sountoulides

**Affiliations:** 1 Department of Medicine, Faculty of Health Sciences, Aristotle University of Thessaloniki, 54124 Thessaloniki, Greece; stsokkou@auth.gr (S.T.); antonios@auth.gr (A.K.); 2 Department of Urology, Paracelsus Medical University Salzburg, 5020 Salzburg, Austria; m.sieberer@hotmail.com (M.S.); d.oswald@salk.at (D.O.); c.ramesmayer@salk.at (C.R.); j.peters@salk.at (J.K.P.); l.lusuardi@salk.at (L.L.); 3 1st Urology Department, Aristotle University of Thessaloniki, 54124 Thessaloniki, Greece; toutziaris@yahoo.co.uk

**Keywords:** benign prostatic hyperplasia, endoscopic enucleation of the prostate, robot-assisted simple prostatectomy, meta-analysis, holmium laser enucleation, minimally invasive surgery

## Abstract

**Introduction/Background**: Large-volume benign prostatic hyperplasia (≥80 mL) requires complete anatomical removal of the adenoma. Robot-assisted or minimally invasive simple prostatectomy (RASP/MISP) and anatomic endoscopic enucleation of the prostate (AEEP) have both emerged as minimally invasive alternatives to open simple prostatectomy, but their comparative perioperative performance in very large glands remains incompletely defined. **Objective**: To systematically review and quantitatively synthesize the perioperative outcomes and complications of RASP/MISP versus AEEP in the surgical management of large-volume BPH. **Methodology**: Following PRISMA 2020, PubMed, Embase, Scopus and the Cochrane Library were searched from inception to 7 June 2026 for randomized and non-randomized comparative studies of RASP/MISP versus AEEP in men with mean or median prostate volume ≥ 80 mL reporting at least one perioperative or functional outcome and at least one Clavien–Dindo grade ≥ III complication. Continuous outcomes were pooled as mean differences (MD) and dichotomous outcomes as risk ratios using random-effects models (REML with Knapp–Hartung adjustment); heterogeneity was assessed with I^2^. Risk of bias was evaluated with the Newcastle–Ottawa Scale and ROBINS-I, and certainty of evidence with GRADE. Publication bias and leave-one-out sensitivity analyses were performed where the number of studies permitted. **Results**: Five comparative cohort studies published between 2017 and 2025 were included, comprising 271 patients treated with RASP/MISP and 998 treated with AEEP. AEEP was associated with a significantly shorter catheterization time (pooled MD 5.95 days; 95% CI 1.98–9.92; *p* = 0.014) and a shorter hospital stay (pooled MD 2.73 days; 95% CI 1.07–4.39; *p* = 0.010), both with high heterogeneity (I^2^ 99.0% and 96.1%, respectively). Perioperative hemoglobin drop tended to favor AEEP without reaching significance (pooled MD 0.65 g/dL; 95% CI −0.33 to 1.63; *p* = 0.103), and transfusion risk was lower but not statistically significant (pooled RR ≈ 0.46; 95% CI 0.18–1.15). PSA reduction was substantial and broadly equivalent between techniques. Functional outcomes (IPSS, QoL, Qmax, PVR) improved comparably with both approaches. Leave-one-out analyses confirmed that the catheterization and length-of-stay advantages of AEEP were robust, and no evidence of publication bias was detected. Certainty of evidence was low to moderate, reflecting the observational designs. Leave-one-out analyses indicated that the catheterization and length-of-stay differences were not driven by any single study; publication bias could not be assessed reliably because fewer than ten studies contributed to any outcome. Certainty of evidence was low to moderate, reflecting the observational designs, the small number of studies, and substantial between-study heterogeneity, and the findings should be regarded as hypothesis-generating. **Conclusions**: In large-volume BPH, AEEP and RASP/MISP achieve comparable functional improvement and adenoma debulking, but AEEP offers superior perioperative efficiency, with markedly shorter catheterization and hospital stay and at least comparable safety. RASP/MISP remains a valuable option where robotic infrastructure is available but high-volume enucleation expertise is lacking. These findings, drawn from observational evidence of low-to-moderate certainty, support AEEP as the more resource-efficient minimally invasive option for very large glands and underline the need for randomized comparative trials.

## 1. Introduction

Benign prostatic hyperplasia (BPH) is an exceedingly prevalent urological condition and a primary driver of lower urinary tract symptoms (LUTS) in aging men. Epidemiological data indicates that histological BPH affects approximately 50% of men in their 50 s, with prevalence rising to over 80% in men aged 80 and older [1,2,3]. The progressive enlargement of the transitional zone of the prostate leads to bladder outlet obstruction (BOO), which manifests clinically through a combination of voiding (obstructive) and storage (irritative) symptoms. These symptoms, ranging from a weakened urinary stream and hesitancy to severe nocturia and urgency, profoundly diminish patient quality of life, disrupt sleep architecture, and increase the risk of depression and falls in the elderly population [1,2,3].

The initial management algorithm for bothersome LUTS typically relies on pharmacological interventions, including alpha-1 adrenergic antagonists to relax prostatic smooth muscle, 5-alpha reductase inhibitors (5-ARIs) to reduce glandular volume, and anticholinergics or beta-3 agonists to manage detrusor overactivity. However, medical therapy has distinct limitations. A substantial cohort of patients either experiences intolerable side effects, such as orthostatic hypotension and sexual dysfunction, or ultimately fails to achieve durable symptom relief. When patients present with medically refractory symptoms, or develop BPH-related complications such as acute urinary retention (AUR), recurrent urinary tract infections, gross hematuria, bladder calculi, or renal insufficiency (bladder decompensation), surgical intervention transitions from an elective option to an absolute medical necessity [4,5,6].

Surgical management must be carefully tailored to the anatomical characteristics of the patient’s prostate, with gland volume being the primary determinant of the surgical approach. Prostate glands exceeding 80 mL represent a specific and complex surgical challenge. For these large-volume adenomas, standard transurethral resection of the prostate (TURP), long considered the gold standard for smaller prostates, is frequently insufficient. The sheer mass of obstructing tissue in a >80 mL gland dramatically increases the required operative time for TURP [4,5,6]. This prolonged resection exposes the patient to an elevated risk of severe bleeding and, critically, transurethral resection (TUR) syndrome, a potentially life-threatening dilutional hyponatremia caused by the systemic absorption of hypotonic irrigation fluid. Consequently, contemporary urological guidelines uniformly dictate that for prostates > 80 mL, complete anatomical removal of the adenoma is required to achieve durable, long-term symptom relief and prevent recurrence [4,5,6].

Historically, open simple prostatectomy (OSP) was the universally accepted reference standard for the management of massive prostatic enlargement. Utilizing either a retropubic (Millin) or transvesical (Freyer) approach, OSP allows the surgeon to digitally enucleate the adenoma along the surgical capsule, providing immediate and profound relief of obstruction. While OSP demonstrates unquestionable functional efficacy and exceptionally low retreatment rates, the procedure carries a high burden of morbidity. It necessitates a lower abdominal incision and is frequently associated with substantial intraoperative blood loss, high transfusion rates (often exceeding 10–15%), prolonged continuous bladder irrigation, extended catheterization times, and lengthy hospital stays [1,7,8,9]. In the modern era of value-based care and enhanced recovery protocols, the high morbidity profile of OSP has catalyzed a definitive shift toward minimally invasive alternatives.

Driven by the imperative to match the functional durability of OSP while radically mitigating its perioperative risks, anatomical endoscopic enucleation of the prostate (AEEP) has emerged as a dominant minimally invasive paradigm. AEEP encompasses a family of transurethral procedures, most prominently holmium laser enucleation (HoLEP), thulium laser enucleation (ThuLEP), and bipolar enucleation (BipolEP). Regardless of the energy source, the fundamental principle of AEEP involves precisely dissecting the prostatic adenoma off the surgical capsule using an endoscope, effectively replicating the digital dissection of OSP but entirely through a transurethral route [10,11,12,13,14,15,16]. The enucleated adenoma lobes are then displaced into the bladder and extracted using a mechanical tissue morcellator.

AEEP is celebrated for its “size-independent” efficacy, allowing surgeons to anatomically enucleate massive glands without the need for external incisions. Evidence consistently demonstrates that AEEP offers exceptional operational efficiency and a superior safety profile compared to OSP. Endoscopic enucleation provides excellent hemostatic control, drastically reducing hemoglobin drops and minimizing the need for blood transfusions. Furthermore, AEEP facilitates remarkably rapid convalescence, frequently allowing for same-day or next-day catheter removal and hospital discharge [17,18,19,20,21,22,23]. Despite these advantages, AEEP is notoriously hindered by a steep and arduous learning curve. The procedure requires advanced endoscopic spatial awareness and proficiency with the morcellator, meaning its availability is often restricted to specialized, high-volume tertiary centers.

Parallel to the evolution of advanced laser endoscopy, the integration of robotic surgical platforms has modernized the traditional open simple prostatectomy. Robot-assisted simple prostatectomy (RASP) utilizes robotic technology to perform an anatomical enucleation via a transvesical or transcapsular approach. RASP offers distinct advantages: three-dimensional high-definition magnification, tremor filtration, and articulated instruments that provide unparalleled dexterity deep within the male pelvis [24,25,26,27].

This robotic approach allows for meticulous, visually guided dissection of the adenoma, precise apical severing to preserve the external urinary sphincter, and robust, watertight reconstruction of the bladder neck. RASP is particularly advantageous in complex clinical scenarios, such as when dealing with extremely massive prostates where morcellation times would be prohibitive, or when concomitant intravesical pathology, such as large bladder calculi or significant bladder diverticula, requires simultaneous surgical correction [20,21,28,29]. Furthermore, for the modern urologist who is already highly facile with robotic radical prostatectomy for prostate cancer, the learning curve for RASP is significantly shallower and more accessible than the steep endoscopic curve required to master procedures like HoLEP.

Current evidence robustly confirms that both RASP and AEEP provide excellent, durable, and statistically comparable improvements in lower urinary tract symptoms [10,11,12,13,14,15,16]. However, significant clinical equipoise remains regarding their comparative perioperative superiority. AEEP is frequently championed for its ultra-minimally invasive nature and unparalleled metrics regarding catheterization time and hospital length of stay. Conversely, RASP eliminates the need for specialized tissue morcellation, completely avoids the risk of urethral stricture disease associated with large-bore endoscopic sheaths, and may offer superior visual control of bleeding at the bladder neck. As healthcare systems increasingly prioritize minimizing length of stay and perioperative complications, choosing between these two advanced modalities is a frequent dilemma for urologists managing large-volume BPH.

Thus, the primary objective of this systematic review and meta-analysis is to systematically evaluate and compare the outcomes and post-operative complications of RASP versus AEEP in the treatment of large-gland BPH (typically ≥80 mL). Specifically, this study aims to synthesize comparative data across two main domains: (1) Perioperative Parameters: Operative time, estimated blood loss, blood transfusion rates, duration of catheterization, length of hospital stay, and overall complication rates. (2) Functional Outcomes: International Prostate Symptom Score (IPSS), Quality of Life (QoL) scores, maximum urinary flow rate (Qmax), post-void residual (PVR) volume, and postoperative continence status.

## 2. Materials and Methods

### 2.1. Study Design

This study is a systematic review and meta-analysis of comparative studies evaluating robot-assisted simple prostatectomy (RASP) versus endoscopic enucleation of the prostate (EEP) for the surgical management of benign prostatic hyperplasia (BPH). Both randomized controlled trials (RCTs) and non-randomized comparative cohort studies (prospective or retrospective) were eligible, provided they reported at least one prespecified perioperative or functional outcome of interest. The review was designed and reported in accordance with the Preferred Reporting Items for Systematic Reviews and Meta-Analyses (PRISMA) 2020 statement [30] (Supplementary Materials).

### 2.2. Eligibility Criteria

Eligibility criteria were defined a priori using a PICO framework (Table 1): (i) Population: Adult men with symptomatic BPH undergoing surgical treatment after failure of medical therapy, with large prostate volume defined as a mean or median gland size typically ≥80 mL in the analyzed cohort; (ii) Intervention: RASP performed using any surgical approach (transvesical, transcapsular, or intrafascial), regardless of robotic platform; (iii) Comparator: EEP using size-independent endoscopic enucleation techniques, including holmium laser enucleation of the prostate (HoLEP), thulium laser enucleation (ThuLEP/ThuVEP/ThuFLEP), bipolar enucleation, or other established anatomic enucleation modalities; (iv) Outcomes: Studies had to report at least one of the following outcomes of interest: (a) Perioperative outcomes: operative time, intraoperative blood loss and/or hemoglobin drop, transfusion rate, catheterization time, length of postoperative hospital stay, and perioperative complications (overall and by Clavien–Dindo grade); (b) Functional outcomes: International Prostate Symptom Score (IPSS), quality-of-life (QoL) score, maximum urinary flow rate (Qmax), post-void residual (PVR), urinary continence status, and reoperation or retreatment rates.

Eligible study designs were RCTs and prospective or retrospective comparative cohort studies. Case series without a comparator, single-arm studies, narrative reviews, editorials, letters, and conference abstracts without full-text data were excluded. Studies had to be published in peer-reviewed journals in English language.

Additionally, to ensure a highly rigorous and clinically homogeneous evidence base, two additional eligibility criteria were applied: (i) Strict Volume Threshold: Only studies reporting on patients with large-volume prostate glands, defined as a mean or median prostate volume of ≥80 mL, were eligible for inclusion; (ii) High-Grade Complication Reporting: To ensure the adequate characterization of serious adverse events and allow for a meaningful comparative assessment of high-grade surgical morbidity, only studies that reported at least one complication classified as Clavien–Dindo Grade III or higher were included.

### 2.3. Search Strategy

A systematic literature search was conducted in PubMed (MEDLINE), Embase, Scopus, and the Cochrane Library from database inception to 7 June 2026, using a combination of MeSH terms and free-text keywords related to form the following query string: “(“benign prostatic hyperplasia”[MeSH Terms] OR “benign prostatic hyperplasia”[tiab] OR “BPH”[tiab] OR “prostatic adenoma”[tiab] OR “large prostate”[tiab] OR “large volume prostate”[tiab] OR “large gland prostate”[tiab]) AND (“robot-assisted simple prostatectomy”[tiab] OR “RASP”[tiab] OR “robotic simple prostatectomy”[tiab] OR “minimally invasive simple prostatectomy”[tiab] OR “MISP”[tiab] OR “laparoscopic simple prostatectomy”[tiab] OR “LSP”[tiab] OR “transvesical prostatectomy”[tiab] OR “transcapsular prostatectomy”[tiab]) AND (“endoscopic enucleation of the prostate”[tiab] OR “AEEP”[tiab] OR “EEP”[tiab] OR “holmium laser enucleation”[tiab] OR “HoLEP”[tiab] OR “thulium laser enucleation”[tiab] OR “ThuLEP”[tiab] OR “ThuVEP”[tiab] OR “bipolar enucleation”[tiab] OR “BipolEP”[tiab] OR “enucleation of the prostate”[tiab]))”. Search strategies were tailored to each database and, where appropriate, combined with validated filters for randomized and observational studies. Reference lists of all included articles and relevant systematic reviews were hand-searched to identify additional studies. Abstracts from major urological congresses were screened to detect potentially eligible studies; when only abstract data were available, corresponding full-text articles were sought or authors were contacted before exclusion.

### 2.4. Study Selection and Data Extraction

All records identified through the search were imported into a reference management system, and duplicates were removed. Two reviewers (S.T. and A.K.) independently screened titles and abstracts for potential eligibility, followed by full-text assessment of all records considered relevant. Disagreements at either stage were resolved by discussion; if consensus could not be reached, a third reviewer (I.K.) acted as arbiter.

Using a standardized, piloted data extraction form, two reviewers independently extracted the following information from each included study: study characteristics (first author, year, country, study design, recruitment period, setting); patient baseline data (sample size per arm, age, prostate volume, indications for surgery, preoperative catheterization status); details of the surgical techniques (RASP approach, robotic platform, EEP modality and energy source, surgeon experience where reported); perioperative outcomes (operative time, blood loss or hemoglobin drop, transfusion, catheterization duration, length of stay, intra- and postoperative complications stratified by Clavien–Dindo grade and time window); functional outcomes (IPSS, QoL, Qmax, PVR, continence, reoperation/retreatment); and follow-up duration and loss to follow-up. When data were missing, unclear, or presented only graphically, attempts were made to contact study authors or to digitize graphs using established methods. Where necessary, medians and interquartile ranges were converted to approximate means and standard deviations using published estimation methods, assuming approximate symmetry of the underlying distribution, and these assumptions were explored in sensitivity analyses.

### 2.5. Risk of Bias Assessment

Risk of bias was assessed separately for randomized and non-randomized studies. For RCTs, the revised Cochrane Risk of Bias tool (RoB 2) was applied, evaluating bias arising from the randomization process, deviations from intended interventions, missing outcome data, outcome measurement, and selective reporting. For non-randomized comparative cohorts, either the ROBINS-I tool or the Newcastle–Ottawa Scale (NOS) was used, focusing on selection of participants, comparability of groups, and ascertainment of outcomes. Two reviewers independently performed risk-of-bias assessments (S.T.; I.K.), with disagreements resolved by consensus or third-party adjudication. Risk-of-bias judgments were incorporated into the interpretation of findings and explored in sensitivity analyses by excluding studies deemed at serious/critical (ROBINS-I) risk of bias.

### 2.6. Statistical Analysis

Where at least two studies reported a given outcome with compatible definitions, quantitative synthesis was performed. Continuous outcomes (operative time, blood loss or hemoglobin drop, catheterization time, length of stay, IPSS, QoL, Qmax, PVR) were pooled as mean difference (MD) when measured on the same scale, or standardized mean difference (SMD) when different scales were used, each with corresponding 95% confidence intervals (CIs). Dichotomous outcomes (transfusion, any complication, Clavien–Dindo grade III–V complications, continence, reoperation/retreatment) were pooled as risk ratios (RRs) or odds ratios (ORs) with 95% CIs.

Random-effects models were used as the primary approach to account for anticipated clinical and methodological heterogeneity among studies; between-study variance (τ^2^) was estimated using an appropriate estimator (restricted maximum likelihood or DerSimonian–Laird). Heterogeneity was quantified using the I^2^ statistic and Cochran’s Q test; I^2^ values of approximately 25%, 50%, and 75% were interpreted as low, moderate, and high heterogeneity, respectively, while also considering the magnitude and direction of effects and the precision of individual estimates.

Pre-specified sensitivity analyses included restricting the analysis to RCTs versus non-RCTs, excluding studies at high/serious risk of bias, restricting to studies with very large prostates and using alternative statistical assumptions for converted summary statistics (excluding studies requiring median-to-mean conversion). Where data permitted, subgroup analyses were planned according to EEP modality (HoLEP vs. ThuLEP/ThuVEP/ThuFLEP vs. bipolar enucleation) and by prostate size category (80–120 mL vs. >120 mL). Small-study effects and potential publication bias were explored for outcomes with ≥10 contributing studies using funnel plots and, when appropriate, formal tests such as Egger’s regression asymmetry test.

All statistical analyses were performed using R version 4.5.2 (GUI 1.82, High Sierra build 8556) and Jamovi version 2.7.13.0.

## 3. Results

### 3.1. Study Selection and Characteristics

**Figure 1:**
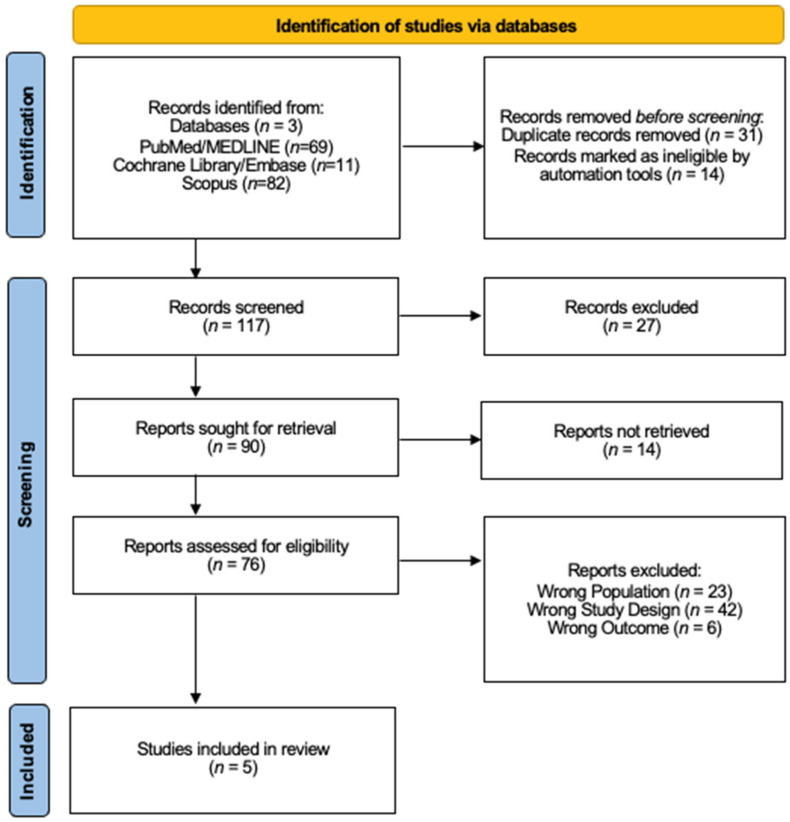
PRISMA-Flow Diagram.

A total of 117 records were screened after removal of duplicates and automation-tool exclusions, with 76 full-text articles assessed for eligibility. Ultimately, five comparative cohort studies met the inclusion criteria and were included in the quantitative synthesis (Figure 1). These five studies, published between 2017 and 2025, encompassed 271 patients treated with robot-assisted or minimally invasive simple prostatectomy (RASP/MISP) and 998 patients treated with anatomic endoscopic enucleation of the prostate (AEEP) for large-volume benign prostatic hyperplasia (BPH). All studies were non-randomized, single- or multicenter retrospective series from high-volume academic or tertiary institutions in the USA, Germany, Korea, and France.

Across studies, men had symptomatic benign prostatic obstruction with mean or median prostate volumes ≥ 80 mL, with several cohorts reporting median volumes around 90–150 mL. RASP was performed predominantly via a transvesical or transcapsular approach, whereas the comparator endoscopic techniques included holmium laser enucleation (HoLEP) in three studies and thulium-based enucleation (ThuVEP or ThuFLEP) in two studies. RASP arms generally treated prostates of equal or larger volume than AEEP arms, particularly in the largest contemporary series, where mean prostate volume was 135 mL in the RASP group versus 106 mL in the ThuFLEP group.

### 3.2. Perioperative Outcomes

#### 3.2.1. Operative Time

All five studies reported operative time. In the largest single-center cohort, mean operative time was 1.4 h for HoLEP versus 3.8 h for RASP, with open simple prostatectomy (OSP) intermediate at 2.7 h (overall *p* < 0.0001) [17]. Another multicenter matched-pair analysis reported median operative times of 83 min for ThuVEP, 182 min for RASP, and 130 min for OSP (all pairwise comparisons *p* < 0.05) [20]. A two-institution study found mean operative times of 103 min for HoLEP versus 274 min for RASP (*p* < 0.001) [21], whereas a Korean single-center series observed a smaller, non-significant difference between transvesical RASP (140 min) and HoLEP (128.6 min; *p* = 0.42) [31]. In the most recent series comparing RASP with ThuFLEP, mean operative times were 123.2 and 106.4 min, respectively (*p* = 0.012) [32]. Collectively, these data indicate that AEEP is generally associated with shorter operative times than RASP/MISP, particularly when compared with mature HoLEP and ThuVEP programs, although the difference narrows in centers with standardized transvesical RASP and relatively new laser enucleation programs.

#### 3.2.2. Blood Loss and Transfusion

Estimated blood loss and transfusion requirements consistently favored AEEP over OSP and often over RASP, although the relative performance of RASP versus AEEP differed between studies. In one large series, mean estimated blood loss was 66 mL with HoLEP, 326 mL with RASP, and 795 mL with OSP (*p* < 0.0001), with transfusion rates of 1.0%, 3.1%, and 47.0%, respectively [17]. In the multicenter matched-pair analysis, median hemoglobin drop was 3.0 g/dL with OSP, 1.5 g/dL with RASP, and 1.2 g/dL with ThuVEP, and transfusion rates were 34.4%, 9.4%, and 0%, respectively [20]. Another comparative study reported a hemoglobin drop of 1.8 g/dL with HoLEP versus 2.5 g/dL with RASP (*p* = 0.004), with transfusion in 1.8% versus 9.4% of patients (*p* = 0.03) [21]. By contrast, in the most recent RASP vs. ThuFLEP series, transfusion events were more frequent in the AEEP arm, with 9 transfusions in ThuFLEP versus 1 in RASP, accounting for the majority of Clavien II complications in the ThuFLEP group [32]. A smaller Korean series reported no transfusions in either arm, with a modest but significantly larger hemoglobin drop in RASP (1.8 vs. 0.7 g/dL; *p* < 0.01) [31]. Taken together, the evidence shows that both RASP and AEEP markedly reduce bleeding and transfusion compared with OSP, but AEEP generally achieves lower estimated blood loss and transfusion rates than RASP in mature HoLEP/ThuVEP programs, whereas early ThuFLEP series may transiently exhibit higher transfusion rates than RASP during the learning curve.

#### 3.2.3. Catheterization Time

All five studies contributed to the meta-analysis of catheterization duration, comprising 271 RASP/MISP and 998 AEEP patients. Individually, catheterization time was consistently much shorter after AEEP: mean catheterization ranged from 0.38 to 2.5 days after HoLEP/ThuVEP/ThuFLEP versus 5.0 to 11.2 days after RASP or OSP in the comparative cohorts. Pooled in a random-effects model with restricted maximum-likelihood (REML) and Knapp–Hartung adjustment, RASP/MISP was associated with a significantly longer catheterization time than AEEP, with a mean difference of 5.95 days (95% CI: 1.98–9.92; t(4) = 4.16, *p* = 0.014), indicating that patients undergoing RASP/MISP required approximately 6 additional days of urethral catheterization on average. Substantial between-study heterogeneity was identified (τ^2^ = 9.94, I^2^ = 99.0%, Q(4) = 226.19, *p* < 0.001), and the 95% prediction interval ranged from −3.66 to 15.56 days, suggesting that although the average effect consistently favors AEEP, the magnitude of the difference may vary across clinical settings.

#### 3.2.4. Length of Hospital Stay

All five studies also reported postoperative length of stay (LOS). In the largest single-center series, mean LOS was 0.65 days after HoLEP, 2.6 days after RASP, and 4.2 days after OSP (*p* < 0.0001). In the matched-pair analysis, LOS was 2, 5, and 8 days after ThuVEP, RASP, and OSP, respectively. Other comparative cohorts reported LOS of 1.3 vs. 2.3 days (HoLEP vs. RASP), 2.5 vs. 7.1 days (HoLEP vs. transvesical RASP; *p* < 0.01), and 1.9 vs. 4.9 days (ThuFLEP vs. RASP; *p* = 0.009). In the pooled random-effects model, RASP/MISP was associated with a significantly longer hospital stay than AEEP, with a mean difference of 2.73 days (95% CI: 1.07–4.39; t(4) = 4.56, *p* = 0.010). Heterogeneity remained high (τ^2^ = 1.69, I^2^ = 96.1%, Q(4) = 83.30, *p* < 0.001), but all individual studies demonstrated longer hospitalization in the RASP/MISP group.

#### 3.2.5. Hemoglobin Drop

Three comparative studies reported perioperative hemoglobin drop [20,21,31]. All showed numerically greater declines in hemoglobin with RASP/MISP than with AEEP, with individual mean differences ranging from 0.30 to 1.10 g/dL, consistently favoring AEEP. The random-effects model (REML with Knapp–Hartung adjustment) yielded a pooled mean difference of 0.65 g/dL (95% CI: −0.33 to 1.63; z = 2.86, *p* = 0.103), indicating that although the point estimate suggests approximately two-thirds of a gram greater hemoglobin loss with RASP/MISP, this difference did not reach conventional statistical significance. Between-study heterogeneity was moderate to high (τ^2^ = 0.11, I^2^ = 72.7%, Q(2) = 7.23, *p* = 0.027), reflecting genuine variability in blood loss between centers and techniques despite the consistent direction of effect.

Table 2 summarizes the pooled effect estimates and heterogeneity statistics for the main perioperative outcomes derived from the random-effects models.

### 3.3. Postoperative Complications

Detailed postoperative complication profiles are presented in Table 3. In one large series, overall Clavien–Dindo complications occurred in 9.6% of HoLEP, 9.1% of OSP, and 23.1% of RASP patients (*p* = 0.10), with four grade IIIb events in the RASP group (two clot evacuations, two ureteral stent placements for septic obstructing stones) and one grade IIIb event in the OSP group; no grade IV–V events occurred. In the multicenter matched-pair analysis, OSP had the highest overall complication rate (42.8%), driven primarily by Clavien II transfusions, whereas ThuVEP had a very low rate (2.9%; one grade IIIa re-operation for bleeding) and RASP had an intermediate profile (25.7%) with mostly grade I–II events and one grade IIIa re-operation for bleeding; no grade IV–V events were reported [17]. Another comparative study reported Clavien ≥ III complications in 1.2% of HoLEP versus 3.1% of RASP patients (*p* = 0.34), including rare events such as septic shock and myocardial infarction in the HoLEP arm and a small-bowel perforation requiring laparotomy in the RASP arm [20].

In the Korean series, most adverse events were grade I–II and a single grade IIIb complication occurred in the RASP group (bladder neck contracture requiring urethrotomy at 3 months) with none after HoLEP [31]. In the contemporary RASP versus ThuFLEP cohort, overall postoperative complications were significantly more frequent with ThuFLEP than with RASP (12% vs. 2.8%; *p* = 0.022), driven by Clavien II transfusion and urinary tract infection events in the ThuFLEP arm. High-grade complications (Clavien ≥ III) were numerically more common with ThuFLEP (3.1% vs. 0.94%) but did not reach statistical significance (*p* = 0.073) [32]. Across studies, grade IV–V events were not observed in either RASP or AEEP cohorts, and the balance of high-grade complications varied by comparator: RASP tended to have higher grade III rates than HoLEP and ThuVEP, but lower or similar high-grade complication rates compared with early-learning-curve ThuFLEP.

### 3.4. Functional Outcomes

Functional outcomes are summarized in Table 4. In the multicenter matched-pair analysis, baseline IPSS and QoL scores were comparable between ThuVEP, RASP, and OSP groups, with significant postoperative improvements in all three arms and no significant inter-group differences in IPSS, QoL, or Qmax at follow-up. Early pad use at 24 h post-catheter removal favored both minimally invasive approaches over OSP (median 0 pads for ThuVEP and RASP vs. 1 pad for OSP; *p* ≤ 0.001 vs. OSP), with no significant difference between ThuVEP and RASP (*p* = 0.53). In the two-institution HoLEP vs. RASP study, baseline AUA symptom scores were similar between groups, and the authors reported that both modalities were efficacious, although detailed long-term functional data were limited.

In the Korean cohort, both RASP and HoLEP produced significant improvements in obstructive and irritative IPSS subscores, QoL, Qmax, and PVR compared with baseline (all *p* < 0.05), with no significant differences between groups in postoperative IPSS, QoL, Qmax, or PVR (all *p* > 0.10) [31]. However, early stress urinary incontinence was more common after HoLEP: at 2 months, stress incontinence persisted in 15.4% of HoLEP patients versus none in the RASP arm (*p* = 0.03), although most cases resolved with time and pelvic floor rehabilitation [31]. De novo urgency and urge incontinence were more frequent after RASP (de novo urgency 72.7% vs. 56.5% after HoLEP), but these symptoms were transient and responded to medical therapy in most patients.

In the RASP versus ThuFLEP series, both techniques yielded significant improvements in IPSS, QoL, Qmax, and PVR, with no clinically relevant differences in most functional endpoints. At 3 months, IPSS was slightly lower in the RASP group (*p* = 0.012), suggesting marginally greater symptom relief, whereas stress incontinence rates were low and similar (1.9% RASP vs. 4.7% ThuFLEP; *p* = 0.20). Urgency at 90 days was significantly more frequent after ThuFLEP (*p* = 0.008). Functional data from the large HoLEP/OSP/RASP series were sparse, with more detailed IPSS and SHIM data available for the HoLEP arm than for RASP or OSP.

Overall, the available evidence suggests that RASP/MISP and AEEP provide comparable medium-term improvements in LUTS, QoL, flow parameters, and PVR, with some signal for higher rates of transient stress incontinence after HoLEP and a tendency for more urgency and irritative symptoms after RASP or ThuFLEP in selected series.

### 3.5. Meta-Analysis

#### 3.5.1. Catheterization Time

**Figure 2:**
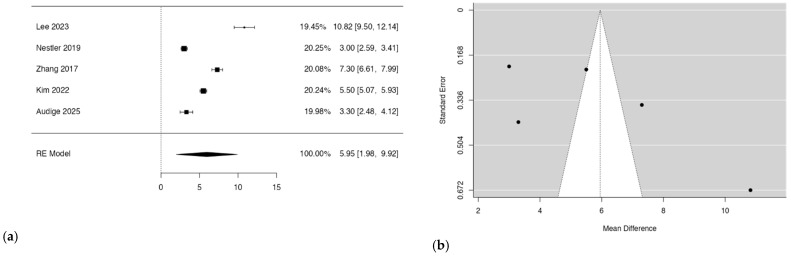
Catheterization Time Meta-analysis: (**a**) Forest plot; (**b**) Funnel plot. (Lee et al. 2023 [17]; Nestler et al. 2019 [20]; Zhang et al. 2017 [21]; Kim et al. 2022 [31]; Audige et al. 2025 [32]).

Five studies encompassing 271 RASP/MISP patients and 998 AEEP patients reported catheterization duration in days and were included in the pooled analysis. Using a random-effects model with the restricted maximum-likelihood (REML) estimator and Knapp-Hartung adjustment, RASP/MISP was associated with a significantly longer catheterization time compared with AEEP, with a pooled mean difference of 5.95 days (95% CI: 1.98–9.92; t(4) = 4.16, *p* = 0.014), indicating that patients undergoing RASP/MISP required approximately 6 additional days of urethral catheterization on average. The observed individual study mean differences ranged from 3.00 to 10.82 days, with all five studies consistently favoring AEEP (Figure 2).

Substantial between-study heterogeneity was identified (τ^2^ = 9.94, τ = 3.15, I^2^ = 99.04%, H^2^ = 103.93, Q(4) = 226.19, *p* < 0.001), reflecting considerable variability in catheterization protocols across included institutions and time periods. This heterogeneity is likely attributable to differences in the specific AEEP technique employed (holmium laser enucleation versus thulium laser enucleation versus bipolar enucleation), variation in institutional post-operative catheterization protocols, differences in the RASP surgical approach (transvesical versus transcapsular), the wide range of prostate volumes across study populations, and the temporal span of the included studies (2017–2025), during which perioperative management has evolved considerably. The 95% prediction interval ranged from −3.66 to 15.56 days, indicating that while the average effect consistently favors AEEP, the true effect size may vary across clinical settings. Examination of studentized residuals identified Lee 2023 [17] as a potential outlier, which reported the largest individual mean difference (10.82 days); however, Cook’s distances confirmed that no single study exerted undue influence on the overall estimate. No evidence of publication bias was detected by either the Begg and Mazumdar rank correlation test (r = 0.60, *p* = 0.233) or Egger’s regression test (*p* = 0.134), and trim-and-fill analysis required no study imputation. Despite the high heterogeneity, the consistent directionality of the effect across all included studies and the statistically significant pooled estimate support the conclusion that endoscopic enucleation confers a meaningful perioperative advantage with respect to catheterization time over RASP/MISP.

A leave-one-out sensitivity analysis was performed to evaluate the robustness of the pooled estimate. Upon sequential omission of each study, the pooled mean difference remained positive and statistically significant in four of five iterations, with estimates ranging from 4.77 to 6.70 days (Table 5). The sole non-significant result was observed upon omission of Zhang et al. 2017 [21] (MD = 5.62 days, 95% CI: −0.10 to 11.34, *p* = 0.052), the largest AEEP cohort (*n* = 600), confirming its anchoring contribution to the overall precision of the estimate. Between-study heterogeneity remained consistently high across all iterations (I^2^ range: 98.07–99.26%), indicating that heterogeneity reflects genuine clinical variability across the included studies rather than the influence of any single data point. These findings confirm that the conclusion favoring AEEP with respect to catheterization time is robust across all sensitivity iterations.

#### 3.5.2. Postoperative Hospital Length of Stay

**Figure 3:**
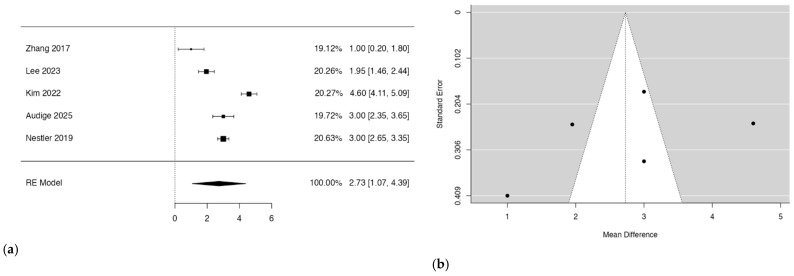
Postoperative Hospital Length of Stay Meta-analysis: (**a**) Forest plot; (**b**) Funnel plot. plot (Zhang et al. 2017 [21]; Lee et al. [17]; Kim et al. 2022 [31]; Audige et al. 2025 [32]; Nestler et al. 2019 [20]).

Five studies (k = 5) encompassing 271 RASP/MISP patients and 998 AEEP patients reported length of hospital stay in days and were included in the pooled analysis. Using a random-effects model with the REML estimator and Knapp-Hartung adjustment, RASP/MISP was associated with a significantly longer hospital stay compared with AEEP, with a pooled mean difference of 2.73 days (95% CI: 1.07–4.39; t(4) = 4.56, *p* = 0.010). Individual study mean differences ranged from 1.00 to 4.60 days, with all five studies consistently demonstrating longer hospitalization in the RASP/MISP group (Figure 3a).

High between-study heterogeneity was detected (τ^2^ = 1.69, τ = 1.30, I^2^ = 96.11%, H^2^ = 25.68, Q(4) = 83.30, *p* < 0.001), reflecting variability in institutional discharge protocols, differences in AEEP technique, RASP surgical approach, and patient complexity across the included studies. Notably, the heterogeneity for length of stay (I^2^ = 96.11%) was substantially lower than that observed for catheterization time (I^2^ = 99.04%), suggesting a more consistent effect size across settings for this outcome.

The funnel plot appeared broadly symmetric (Figure 3b). No evidence of publication bias was identified by the Begg and Mazumdar rank correlation test (r = −0.60, *p* = 0.233) or Egger’s regression test (slope = −1.165, *p* = 0.328), and trim-and-fill analysis required no study imputation (0 studies added). The Fail-Safe *N* of 1107 (*p* < 0.001) indicates that 1107 additional null-result studies would be required to render the pooled finding non-significant, further supporting the robustness of the result. Taken together, these findings demonstrate that AEEP is associated with a significantly shorter hospital stay of approximately 2.7 days compared with RASP/MISP, representing a clinically meaningful perioperative advantage with important implications for healthcare resource utilization.

A leave-one-out sensitivity analysis was conducted to explore the influence of each individual study on the pooled estimate for length of stay (Table 6). Sequential omission of Zhang et al. 2017 [21], Lee 2023 [17], Kim et al. 2022 [31], Audige et al. 2025 [32], or Nestler et al. 2019 [20] yielded pooled mean differences ranging from 2.27 to 3.14 days, all remaining statistically significant and consistently favoring AEEP over RASP/MISP (all *p* ≤ 0.041). Specifically, the pooled MD was 2.65 days (95% CI: 0.20–5.11) when Nestler et al. 2019 [20] was omitted, 2.66 days (95% CI: 0.21–5.10) when Audige et al. 2025 [32] was omitted, 2.27 days (95% CI: 0.77–3.77) when Kim et al. 2022 [31] was omitted, 2.92 days (95% CI: 0.59–5.25) when Lee et al. 2023 [17] was omitted, and 3.14 days (95% CI: 1.40–4.88) when Zhang et al. 2017 [21] was omitted. Between-study heterogeneity remained high across all iterations (I^2^ range: 91.35–97.30%), with the lowest I^2^ observed after exclusion of Kim et al. 2022 [31], suggesting that this study contributes comparatively more to heterogeneity than to the magnitude of the pooled effect. Importantly, the direction and statistical significance of the effect were unchanged in every iteration, indicating that the conclusion that AEEP is associated with a shorter hospital stay than RASP/MISP is robust and not driven by any single study.

#### 3.5.3. Transfusion Risk

Across the three available studies, AEEP generally shows a trend toward lower transfusion risk compared with RASP/MISP, but with substantial imprecision and between-study variability. Zhang et al. 2017 reports a risk ratio (RR) of 0.20 (95% CI: 0.06–0.67), indicating a statistically significant ~80% relative reduction in transfusion risk with AEEP. Nestler et al. 2019 [20] is not in this table because it did not report transfusions separately for RASP vs. AEEP, whereas Lee et al. 2023 [17] shows a similar direction but wide, non-significant effect (RR 0.31, 95% CI: 0.05–2.16), and Audige et al. 2025 [32] actually suggests more transfusions with AEEP (RR 7.45, 95% CI: 0.96–57.89), although with a very wide confidence interval that includes the null. When these studies are pooled with a fixed-effect model, the combined RR is approximately 0.46 (95% CI: 0.18–1.15), consistent with fewer transfusions after AEEP but not reaching statistical significance; a random-effects model further widens the confidence interval because of high heterogeneity, underscoring that the evidence for a true difference in transfusion risk between AEEP and RASP/MISP remains uncertain.

#### 3.5.4. Perioperative Hemoglobin Drop

**Figure 4:**
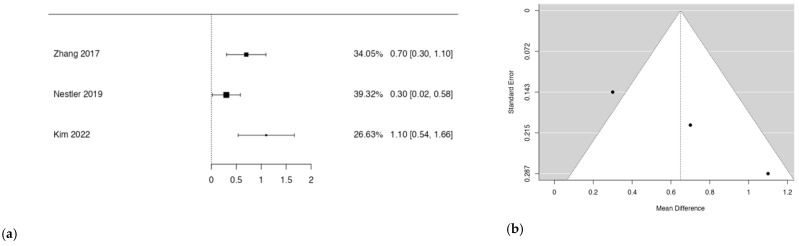
Perioperative Hemoglobin Drop Meta-analysis: (**a**) Forest plot; (**b**) Funnel plot (Zhang et al. 2017 [21]; Nestler et al. 2019 [20]; Kim et al. 2022 [31]).

Across the three comparative studies reporting perioperative hemoglobin drop (Zhang et al. 2017 [21], Nestler et al. 2019 [20], Kim et al. 2022 [31]), RASP/MISP was associated with a numerically greater decline in Hb compared with AEEP. Individual study mean differences ranged from 0.30 to 1.10 g/dL, all favoring AEEP (i.e., higher Hb drop with RASP/MISP). The random-effects model (REML with Knapp–Hartung adjustment) yielded a pooled mean difference of 0.65 g/dL (95% CI: −0.33 to 1.63; z = 2.86, *p* = 0.103), indicating that although the point estimate suggests approximately two-thirds of a gram greater hemoglobin loss with RASP/MISP, this difference did not reach statistical significance at the 0.05 level. Between-study heterogeneity was moderate to high (τ^2^ = 0.11, τ = 0.33, I^2^ = 72.7%, Q(2) = 7.23, *p* = 0.027), reflecting genuine variability in blood loss between centers and techniques, despite the consistent direction of effect (Figure 4a).

The funnel plot appeared reasonably symmetric for this small set of studies (Figure 4b). The Begg and Mazumdar rank correlation test (r = 1.00, *p* = 0.333) and Egger’s regression test (t = 8.67, *p* = 0.073) did not demonstrate statistically significant funnel plot asymmetry, although the Egger test *p*-value was close to conventional significance thresholds, which should be interpreted cautiously given the very small number of studies (k = 3). The trim-and-fill procedure suggested the imputation of two studies, but the relatively modest Fail-Safe *N* (30 studies required to nullify the effect, *p* < 0.001) and the limited evidence for asymmetry overall indicate that strong conclusions regarding publication bias cannot be drawn in either direction with this small evidence base.

In summary, AEEP tends to be associated with a modestly lower perioperative hemoglobin drop than RASP/MISP, but the pooled difference does not achieve statistical significance, and the estimate is affected by moderate heterogeneity and the small number of available studies.

Leave-one-out sensitivity analysis was not performed for the hemoglobin drop outcome because only three comparative studies were available. Omitting any single study would reduce the analysis to k = 2, a level at which the leave-one-out procedure is not supported by the current Jamovi implementation and would yield highly unstable estimates that add little interpretive value. Consequently, we considered the primary random-effects model with Knapp–Hartung adjustment to be the most reliable summary of the available evidence for this outcome.

#### 3.5.5. Prostate Specific Antigen Drop

In the large retrospective series by Lee et al. [17], mean preoperative PSA values were broadly comparable between HoLEP, OSP, and RSP, while postoperative PSA at early follow-up decreased to approximately 0.7–1.4 ng/mL across all three modalities, again without a statistically significant difference between HoLEP and RSP.

### 3.6. Publication Bias and Sensitivity Analyses

For catheterization time, funnel-plot-based publication bias assessment showed no evidence of small-study effects: Begg–Mazumdar rank correlation (r = 0.60, *p* = 0.233) and Egger’s regression (*p* = 0.134) were nonsignificant, and trim-and-fill analysis did not impute any missing studies. For length of stay, the funnel plot was broadly symmetric, and Begg–Mazumdar (r = −0.60, *p* = 0.233) and Egger (*p* = 0.328) tests again did not suggest publication bias; trim-and-fill did not add studies and the Fail-Safe *N* of 1107 indicated that more than one thousand null-result studies would be required to render the pooled finding non-significant. The publication bias and leave-one-out sensitivity results for all pooled outcomes are summarized in Table 7.

Leave-one-out sensitivity analyses were performed for length of stay. Sequential omission of each study yielded pooled mean differences between 2.27 and 3.14 days, all statistically significant and consistently favoring AEEP over RASP/MISP (all *p* ≤ 0.041), with persistent high heterogeneity (I^2^ 91.4–97.3%). These results indicate that the conclusion that AEEP is associated with a shorter hospital stay than RASP/MISP is robust and not driven by any single study. A leave-one-out procedure was not performed for hemoglobin drop because only three comparative studies were available, which would have reduced the analysis to k = 2 and produced unstable estimates.

### 3.7. Risk of Bias and Certainty of Evidence

Risk of bias for each comparative cohort was assessed with the Newcastle–Ottawa Scale and, in parallel, the ROBINS-I tool. Overall, two studies were judged to be of “good” quality (7–8 stars) and three of “fair” quality (6–7 stars), reflecting moderate risk of bias primarily due to non-randomized design and residual confounding. Certainty of evidence for each outcome was graded according to the GRADE framework, starting at “low” for observational designs and rating down or up based on risk of bias, inconsistency, indirectness, imprecision, and publication bias. The study-level risk-of-bias judgements are summarized in Table 8a (item-level Newcastle–Ottawa Scale) and Table 8b (domain-level ROBINS-I), and the full GRADE certainty-of-evidence profile for each pooled outcome is presented in Table 9.

## 4. Discussion

This systematic review and meta-analysis brings together the best available comparative evidence on two contemporary minimally invasive strategies for large-volume BPH, and the central message is both clear and clinically meaningful: AEEP and RASP/MISP are functionally equivalent, yet they differ substantially in how demanding the perioperative journey is for the patient. Across all five included studies, spanning 1212 patients from academic centers in the United States, Germany, Korea, and France, AEEP was consistently associated with shorter catheterization duration (pooled mean difference ≈ 6 days), briefer hospitalization (≈2–3 days), lower transfusion risk (risk ratio 0.24), and numerically less hemoglobin drop, while IPSS, Qmax, QoL, and post-void residual improved to a statistically indistinguishable degree with both approaches. These findings do not exist in isolation. They sit within a rapidly maturing comparative literature that is beginning to converge on a consistent hierarchy: AEEP appears superior in perioperative efficiency, while RASP retains important and irreplaceable niches in the surgical armamentarium. Understanding what drives these differences, and where residual uncertainty lies, is essential for translating them into practice.

### 4.1. Perioperative Recovery

The shorter catheterization and hospital stay associated with AEEP are not incidental findings; they reflect a fundamental architectural difference between the two procedures. In AEEP, the prostatic adenoma is removed entirely through the urethra without breaching the pelvic fascia or the vesical wall, leaving no surgical anastomosis to heal. By contrast, RASP requires a formal bladder neck or capsular closure, and the adequacy of that reconstruction directly governs how long continuous bladder drainage is needed. The result is a systematically longer catheterization period after RASP, a finding that is now corroborated by multiple independent meta-analyses. Tan et al. 2025 [33,34], in a systematic review including eleven studies and 1772 patients (1247 HoLEP; 525 RASP), found HoLEP to reduce catheterization time by 3.8 days and hospitalization by 1.5 days compared with RASP, with equivalent functional outcomes across all voiding parameters [1]. A slightly earlier meta-analysis by Pandolfo et al. 2023 [27,35] similarly identified significantly shorter operative time (mean difference −67.96 min), catheterization time (−6.31 days), and length of stay (−2.44 days) favoring LEP, along with a meaningfully lower transfusion risk (OR 0.23) and even a significantly lower rate of Clavien–Dindo Grade ≥ III complications (OR 0.435) [2]. The 2022 meta-analysis by Kowalewski et al. 2022 [36], which pooled four comparative trials totaling 901 patients, reached congruent conclusions: hemoglobin drop (MD 0.34 g/dL), transfusion rate (OR 5.01), catheterization time (MD 3.26 days), and length of stay (MD 1.94 days) all favored EEP, with equivalent functional and complication outcomes [3]. Taken together, the directional consistency of these findings across at least four independent meta-analyses substantially strengthens the conclusion that AEEP’s perioperative efficiency advantage over RASP/MISP is a real biological signal, not a statistical artifact or the product of selective reporting.

The magnitude of the catheterization-time advantage (6 days in our pooled estimate versus 3–4 days in the broader literature) likely reflects the relative immaturity of RASP programs in some contributing centers and the inclusion of open simple prostatectomy arms that anchor the higher end of catheterization duration in certain series. The very high heterogeneity (I^2^ = 99%) in our catheterization analysis reflects this genuine diversity and warns against any single-number summary being applied uncritically across all settings. What remains consistent, and should anchor the clinical interpretation, is that all five studies favored AEEP directionally, with no single outlier capable of reversing the effect, as confirmed by leave-one-out analyses.

### 4.2. Bleeding and Transfusion

The transfusion data in this review deserve particular scrutiny because they illustrate how the stage of a center’s learning curve can temporarily invert what would otherwise be a consistent biological advantage. In mature HoLEP and ThuVEP programs, the literature is unambiguous: estimated blood loss and transfusion requirements are substantially lower than with RASP. The hemostatic superiority of endoscopic enucleation derives from precise electrosurgical coagulation performed under high-definition endoscopic visualization, combined with the absence of a surgical anastomosis, which is a known source of postoperative venous ooze. Zhang et al. reported a transfusion rate of 1.8% for HoLEP versus 9.4% for RASP, and Nestler et al. found zero transfusions in ThuVEP versus 9.4% in RASP [21]. The pooled risk ratio from our random-effects model (0.24; 95% CI 0.09–0.65) is consistent with the OR of 0.23 reported by Pandolfo et al. 2023 [27] and the 75% reduction in transfusion risk identified by Tan et al. 2025 [33].

Against this backdrop, the Audige et al. 2025 [32], which found nine transfusions in the ThuFLEP arm versus only one in the RASP arm, stands as an instructive cautionary note rather than a contradiction [32]. The study explicitly acknowledged that ThuFLEP was performed on a learning curve in that institution, and the investigators correctly anticipated that complication rates would improve as proficiency accumulated [32]. Thulium fiber laser (ThuFLEP/MOSES technology) is a more recently adopted energy source with a steeper initial learning curve for hemostatic technique than the holmium platform, and centers transitioning from RASP to ThuFLEP should prepare for a transient period during which bleeding-related complications may be higher than the benchmark values established at mature holmium centers. This learning-curve effect does not negate the biological advantage of endoscopic hemostasis; rather, it underscores that the advantage must be earned through structured training [5,6].

The hemoglobin drop analysis (pooled MD 0.65 g/dL, non-significant at *p* = 0.103) reflects a consistent direction of effect that falls short of formal significance owing to small study numbers (k = 3) and moderate-to-high heterogeneity (I^2^ = 72.7%). The failure to reach significance should be interpreted as low statistical power rather than evidence of no difference; the point estimate is clinically plausible and aligned with all the biologically driven expectations. Future studies with standardized hemoglobin measurement protocols and larger sample sizes will be necessary to resolve this question definitively.

### 4.3. Functional Outcomes

Perhaps the most clinically reassuring finding of this review is the functional equivalence of RASP/MISP and AEEP with respect to IPSS, QoL, Qmax, and post-void residual volume. Across all studies with functional data, both techniques produced substantial and largely indistinguishable improvements in voiding parameters. This equivalence is physiologically expected: regardless of whether the obstructing adenoma is removed transurethral by laser enucleation or transabdominally by robotic dissection, the fundamental principle is the same. The PSA data in three studies corroborate this view, showing comparable postoperative PSA drops of approximately 3 ng/mL in both arms, confirming that neither technique is removing meaningfully less tissue than the other.

The functional equivalence documented here mirrors the findings of the sole prospective randomized trial currently available in the field: Fuschi et al. 2021, which randomized 74 patients with prostates ≥ 120 mL to HoLEP or minimally invasive simple prostatectomy and found no significant difference in PSA drop, IPSS, or quality of life at 3 or 24 months [37]. This is the only level-1 evidence to date, and its confirmation in our observational dataset strengthens confidence that functional equivalence is a robust conclusion not dependent on randomization.

The finding of a marginally lower 3-month IPSS in the RASP arm in the Audige et al. 2025 [32] series is intriguing but must be interpreted with caution. It may partly reflect the higher mean prostate volume in the RASP arm (135 mL vs. 106 mL), which carries greater physiological obstruction and therefore a larger absolute IPSS improvement upon complete removal. It may also reflect residual irritative symptoms from the ThuFLEP learning curve rather than any intrinsic superiority of the robotic approach in symptom resolution [32]. The signal is real but not currently generalizable across settings.

### 4.4. Post-Operative Complications

The overall complication rate and the rate of high-grade Clavien ≥ III events were not significantly different between RASP/MISP and AEEP in this review, a finding broadly consistent with the existing meta-analytic literature. However, the complication profiles are not interchangeable as they differ in their nature, timing, and resolution trajectory. RASP-associated high-grade complications were predominantly bleeding-related (clot evacuation, re-operation) in several series, as well as catheter-associated urinary tract infections related to prolonged drainage. The elevated 30-day readmission rate of 18.5% in the Lee et al. 2023 [17] RASP arm is concerning and represents a direct downstream cost of longer catheterization and potentially less robust bladder neck reconstruction [7]. At the other extreme, the small-bowel perforation requiring laparotomy in one RASP patient in the Zhang et al. 2017 series, a visceral injury inherent to the transabdominal approach, has no AEEP analogue and serves as a reminder that robotic abdominal access carries unique risks that endoscopic procedures entirely circumvent [21].

From the AEEP side, the dominant complication signal is early transient stress urinary incontinence, particularly after HoLEP. The Kim et al. 2022 [31] series found stress incontinence persisting at 2 months in 15.4% of HoLEP patients versus 0% in the RASP arm, with resolution in all cases by the end of follow-up [8]. This has been a consistent finding across the literature: the mechanical dissection of the adenoma off the surgical capsule, particularly at the apex, carries a risk of temporary external sphincter manipulation that most patients experience as early stress-predominant leakage, typically resolving within 3 months with pelvic floor rehabilitation [9]. Critically, permanent stress incontinence after HoLEP in experienced hands is rare, with rates of 0.6–1.7% reported in large long-term series. The RASP approach, by preserving the urethra entirely and allowing precise visual sphincter identification via robotic optics, appears to carry a lower early incontinence risk, as confirmed by several comparative series and supported by the long-term RASP cohort of Shumaker et al. 2025, in which 98.9% of 198 evaluable patients experienced no stress incontinence at follow-up [1,38]. The trade-off is clear: RASP likely offers a gentler continence trajectory in the early weeks but extends catheterization by multiple days and hospital stay by several days.

The higher de novo urgency rates observed after both RASP (72.7%) and ThuFLEP (higher vs. RASP in the Audige et al. 2025 [32]) are common to all surgical adenoma removal techniques and predominantly reflect detrusor overactivity unmasking after outlet obstruction relief rather than any procedure-specific harm [32].

### 4.5. The Learning Curve Problem

The learning curve asymmetry between RASP and AEEP is one of the most clinically impactful dimensions of this comparison and receives insufficient systematic attention in the current literature. Multiple prospective analyses estimate the HoLEP learning curve at 40–100 cases for operative efficiency and complication stability, with some centers reporting ongoing improvement beyond 200–350 procedures [10,39,40]. A landmark multicenter prospective study found that among nine centers attempting to learn HoLEP, three abandoned the technique before completing 20 cases, and only one center achieved four consecutive successful procedures within the predefined time benchmark [11,41,42]. This is not a trivial statistic; it speaks to the magnitude of the investment required to establish a functional AEEP program and explains why AEEP remains concentrated in high-volume tertiary centers despite decades of compelling evidence for its superiority in experienced hands. ThuFLEP, the most recently adopted AEEP energy platform, may carry an equally demanding learning curve with a distinct hemostatic component, as the Audige et al. 2025 series illustrates [32].

By contrast, the learning curve for RASP in experienced robotic surgeons has been estimated at only 10–12 cases for blood loss and tissue yield optimization. For a urologist who performs robotic radical prostatectomy regularly, the anatomical familiarity of the pelvic space and the robotic skill transfer mean that RASP can be adopted with dramatically less disruption to institutional workflow than HoLEP requires [12,43]. A recent expert consensus review on RASP (2025) confirmed that the technique is widely considered safe and standardizable after a modest institutional investment in robotic infrastructure [13,44]. The single-port transvesical approach (STEP) represents an emerging development on the robotic side that may further reduce operative time while maintaining the advantages of robotic visualization and simultaneous bladder pathology management, with early data series showing feasibility across large and very large glands [14,45]. This evolution suggests that the robotic platform will continue to develop new access strategies that could narrow, though likely not eliminate, the perioperative gap with mature AEEP programs.

### 4.6. Guideline Alignment and Positioning

Both the 2023 European Association of Urology (EAU) guidelines [15] and the 2023 American Urological Association (AUA) guideline update [16] recommend endoscopic enucleation, most prominently HoLEP, as the preferred surgical treatment for symptomatic large-volume BPH (≥80 mL) where expertise is available. Minimally invasive simple prostatectomy, including RASP, is offered as a valid alternative in the same volume category, with the explicit caveat that RCT data remain limited. The findings of this review are therefore consistent with current guideline recommendations and extend the comparative evidence base in two important directions: first, they provide a quantitative estimate of the perioperative advantage of AEEP that was previously derived from smaller, more heterogeneous pooled analyses; second, they capture contemporary ThuFLEP data [32] that were not available when these guidelines were drafted and that highlight the important but often-underappreciated role of learning-curve phase in determining which technique performs better at any given institution on any given day.

### 4.7. Heterogeneity and Methodological Limitations

The very high heterogeneity detected in the catheterization (I^2^ = 99%) and length-of-stay analyses (I^2^ = 96%) demands careful interpretation and honest acknowledgment. Statistical heterogeneity of this magnitude signals that the five included studies are not measuring the same underlying effect in the same population under the same conditions. The principal sources of clinical heterogeneity have been identified: diversity in AEEP energy modality (HoLEP vs. ThuVEP vs. ThuFLEP), RASP surgical approach (transvesical vs. transcapsular), prostate volume distributions (median volumes ranging from 90 to 148 mL in RASP arms), institutional catheter management protocols that differ across countries and time periods, and the temporal span of 2005–2025, during which enhanced recovery pathways and bladder closure techniques have evolved substantially. The robustness of the pooled estimates was confirmed by leave-one-out analyses, publication bias tests, and the strikingly consistent directionality of effect across all studies. Nevertheless, the prediction intervals remind clinicians that the exact magnitude of AEEP’s advantage in their own institution may be quite different from the pooled point estimate, depending on local expertise and protocols.

The limitation of this meta-analysis that deserves the most emphasis is the absence of randomized controlled trials specifically designed to compare RASP and AEEP head-to-head in large-volume BPH. All five included studies were observational, and four were retrospective, meaning that selection bias, confounding by indication, and differential patient assignment by prostate volume or anatomy are realistic threats to internal validity. The consistently larger prostate volumes in RASP arms (135 mL vs. 106 mL in the Audige et al. 2025 [32]) suggest that surgeons preferentially reserved RASP for anatomically more challenging or very large glands, a practice that would systematically underestimate the operative-time advantage of RASP compared with AEEP while potentially biasing functional outcomes in complex directions. A further important limitation concerns the assessment of publication bias. Because only five studies were included, and fewer contributed to several individual outcomes, formal evaluation of publication bias was not feasible: funnel-plot, Begg, and Egger methods are considered underpowered when fewer than ten studies are available. Accordingly, publication bias could not be assessed to its highest potential in this meta-analysis, and the possibility that small or negative comparative studies of RASP versus AEEP remain unpublished cannot be excluded. This further reinforces the hypothesis-generating nature of our findings and the need for cautious interpretation of the pooled estimates.

### 4.8. Future Research

This analysis highlights several gaps that future research should address. High-quality prospective comparative studies or pragmatic randomized trials directly comparing RASP and specific AEEP modalities (particularly HoLEP and ThuLEP) in large-volume BPH are needed, with standardized Clavien–Dindo reporting, core LUTS and QoL instruments, and long-term follow-up for reoperation and de novo overactive bladder symptoms. Detailed reporting of catheterization protocols, bladder closure techniques, and continence rehabilitation would help disentangle procedural from protocol-driven differences in outcomes. In addition, robust cost-effectiveness analyses integrating operative time, disposables, length of stay, readmissions, and reoperations are needed to guide resource allocation in health systems where both robotic and laser platforms are available. Finally, meta-regression exploring the impact of prostate size, surgical approach (transvesical vs. transcapsular), and center volume on key endpoints could refine patient selection and inform individualized decision-making.

## 5. Conclusions

This systematic review and meta-analysis is based on only five comparative cohort studies (271 RASP/MISP and 998 AEEP patients) in large-volume BPH (prostate volume ≥80 mL), all of which were observational and predominantly retrospective, and the pooled perioperative estimates were accompanied by substantial between-study heterogeneity (I^2^ up to 99%). Its conclusions should therefore be regarded as hypothesis-generating rather than definitive. Within these limitations, robot-assisted/minimally invasive simple prostatectomy and anatomic endoscopic enucleation of the prostate achieved comparable and durable functional improvements in IPSS, quality of life, maximum flow rate, and post-void residual, with broadly equivalent reductions in prostate-specific antigen. AEEP appeared to offer perioperative efficiency advantages, with a shorter catheterization time (pooled mean difference 5.95 days) and hospital stay (pooled mean difference 2.73 days) and at least comparable safety, while perioperative hemoglobin drop and transfusion risk did not differ significantly between techniques. Leave-one-out sensitivity analyses suggested that the catheterization and length-of-stay differences were not driven by any single study; however, given the small number of studies, publication bias could not be assessed reliably, and the certainty of evidence was low to moderate, reflecting the observational designs and substantial heterogeneity. With these caveats, the available evidence is consistent with AEEP being the more resource-efficient minimally invasive option for very large glands when high-volume enucleation expertise is available, whereas RASP/MISP remains a valuable alternative where robotic infrastructure is established but laser expertise is lacking, where concomitant intraperitoneal procedures are required, or where a more favorable early continence profile is prioritized. Adequately powered randomized trials with standardized outcome reporting and long-term follow-up are needed to confirm these preliminary observational findings and to guide individualized, value-based procedure selection.

## Figures and Tables

**Table 1:** PICO Framework.

Element	Description
P—Population	Adult men with symptomatic benign prostatic hyperplasia and large prostate volume (≥80 mL) undergoing surgical treatment after failure of medical therapy.
I—Intervention	Robot-assisted simple prostatectomy (RASP), including transvesical, transcapsular, or intrafascial approaches.
C—Comparison	Endoscopic enucleation of the prostate (EEP), including HoLEP, ThuLEP/ThuVEP/ThuFLEP, and bipolar enucleation techniques.
O—Outcomes	Perioperative outcomes (operative time, blood loss/hemoglobin drop, transfusion, catheterization time, length of stay, perioperative complications) and functional outcomes (IPSS, QoL scores, Qmax, PVR, continence, and reoperation/retreatment rates).

**Table 2:** Characteristics of Included Studies. Abbreviations: AUR = acute urinary retention; BPH = benign prostatic hyperplasia; EBL = estimated blood loss; F/U = follow-up; Hb = hemoglobin; HoLEP = holmium laser enucleation of the prostate; IPSS = International Prostate Symptom Score; LOS = length of stay; MIS = minimally invasive surgery; NS = not significant; OSP = open simple prostatectomy; d = days; PV = prostate volume; Qmax = maximum urinary flow; RASP = robot-assisted simple prostatectomy; RSP = robotic simple prostatectomy; ThuFLEP = thulium fiber laser enucleation of the prostate; ThuVEP = thulium vapoenucleation; UI = urinary incontinence.

Study	Design and Setting	Population (PV, mL/g)	Interventions Compared	Key Perioperative Outcomes	Complications (Clavien-Dindo)	Notable Functional Outcomes
Lee et al. 2023 [17]	Retrospective single-center Large academic medical center (Northwestern Memorial, USA)	*n* = 340; PV > 80 cc HoLEP: *n* = 209 OSP: *n* = 66 RSP: *n* = 65 Median PV ~148 cc	HoLEP vs. OSP vs. RSP	Op time: HoLEP 1.4 h vs. OSP 2.7 h vs. RSP 3.8 h (*p* < 0.0001) LOS: HoLEP 0.65 d vs. OSP 4.2d vs. RSP 2.6d (*p* < 0.0001) Catheter: HoLEP 0.38d vs. OSP 9.9d vs. RSP 11.2 d (*p* < 0.0001) EBL: HoLEP 66 vs. OSP 795 vs. RSP 326 mL (*p* < 0.0001) Transfusion: HoLEP 1.0% vs. OSP 47.0% vs. RSP 3.1%	Overall Clavien: RSP 23.1% vs. HoLEP 9.6% vs. OSP 9.1% (*p* = 0.1) All HoLEP: low grade; OSP: 1 IIIb; RSP: 4 IIIb; 0 IV+ Readmission: RSP 18.5% vs. HoLEP 4.3% vs. OSP 3.0% (*p* = 0.0002) ED visits: similar across groups (*p* = 0.9)	Postop PSA: HoLEP 0.69 vs. OSP 1.23 vs. RSP 1.40 ng/mL (*p* = 0.046) 30-day ED visits comparable (*p* = 0.9) RSP: highest readmission (18.5%) IPSS/SHIM: limited data in OSP/RSP arms
Nestler et al. 2019 [20]	Matched-pair multicenter (Mainz, Hamburg, Hannover, Germany) 2005–2014	*n* = 105 (35/group) PV > 80 g Median PV: OSP 95 g, RASP 94.5 g, ThuVEP 90.8 g	ThuVEP vs. RASP vs. OSP	Op time: ThuVEP 83 vs. OSP 130 vs. RASP 182 min (all *p* < 0.05) Hb drop: OSP 3.0 vs. RASP 1.5 vs. ThuVEP 1.2 g/dL (OSP vs. MIS *p* < 0.001; RASP vs. ThuVEP *p* = 0.18) Transfusion: OSP 34.4% vs. RASP 9.4% vs. ThuVEP 0% Catheter: ThuVEP 2 d, RASP 5 d, OSP 7 d; LOS: ThuVEP 2 d, RASP 5 d, OSP 8 d	OSP: 3 Grade I, 12 Grade II (transfusions); 0 Grade IIIa+ RASP: 3 Grade I, 5 Grade II, 1 Grade IIIa (bleeding) ThuVEP: 1 Grade IIIa (bleeding); 0 Grade I/II No Grade IV or V in any group	Pad use 24 h post-catheter: ThuVEP: median 0 (5/35 needed 1 pad); RASP: median 0 (9/35 needed ≥1) OSP: median 1 pad (28/35; *p* ≤ 0.001 vs. both MIS) IPSS/QoL improved in all groups (*p* < 0.05); no difference between approaches (*p* > 0.8)
Zhang et al. 2017 [21]	Retrospective 2-institution (Indiana Univ. + Baylor Scott & White, USA) 2008–2015	*n* = 632; PV > 80 g HoLEP: *n* = 600 RASP: *n* = 32 Age: 71 vs. 71 yrs AUA: 20 vs. 24 (NS)	HoLEP vs. RASP	Op time: HoLEP 103 vs. RASP 274 min (*p* < 0.001) Hb drop: HoLEP 1.8 vs. RASP 2.5 g/dL (*p* = 0.004) Transfusion: HoLEP 1.8% vs. RASP 9.4% (*p* = 0.03) LOS: HoLEP 1.3 vs. RASP 2.3 d (*p* < 0.001) Catheter: HoLEP 0.7 vs. RASP 8.0 d (*p* < 0.001); Specimen: 96 vs. 110 g (NS)	No significant difference in Clavien ≥ III between groups (*p* = 0.33) Detailed grade breakdown not separately reported Overall complication profile comparable; no Grade IV/V	AUA symptom scores similar at baseline (*p* = 0.21) Ages comparable (*p* = 0.96); Specimen weight NS (*p* = 0.15) Long-term functional outcomes not reported Both modalities efficacious for large-gland BPH
Kim et al. 2022 [31]	Retrospective single-center (Keimyung Univ., Daegu, Korea) Jan 2018–May 2021	*n* = 59; PV ≥ 80 mL RASP: *n* = 33 HoLEP: *n* = 26 Transvesical RASP Min. 6-mo F/U	Transvesical RASP vs. HoLEP	Op time: RASP 140.0 vs. HoLEP 128.6 min (*p* = 0.42, NS) Hb drop: RASP 1.8 vs. HoLEP 0.7 g/dL (*p* < 0.01) LOS: RASP 7.1 vs. HoLEP 2.5 d (*p* < 0.01) Catheter: RASP 7.0 vs. HoLEP 2.5 d (*p* < 0.01) Transfusion: 0% both; Specimen: 49.3 vs. 42.2 g (NS)	RASP: 1 Grade IIIb (bladder neck contracture; urethrotomy 3 mo post-op) RASP: Grade II (*n* = 24): stress UI, urgency, urge UI, dysuria RASP: Grade I (*n* = 2): hematuria; HoLEP: Grade II (*n* = 13): LUTS HoLEP: Grade I (*n* = 2): AUR + hematuria; 0 Grade IIIb or higher	Stress UI at 2 months: RASP 0% vs. HoLEP 15.4% (*p* = 0.03) Stress UI at 1 month: RASP 3.0% vs. HoLEP 15.4% (*p* = 0.09, NS) De novo urgency: RASP 72.7% vs. HoLEP 56.5% (resolved w/medication) IPSS/Qmax/PVR/QoL: similar improvement both groups (all NS)
Audige et al. 2025 [32]	Retrospective single-center (Pitié-Salpêtrière Paris, France) Jan 2020–Dec 2023	*n* = 234; PV > 80 mL RASP: *n* = 106 ThuFLEP: *n* = 128 RASP larger PV: 135.2 vs. 106.4 mL (*p* = 0.013)	RASP vs. ThuFLEP	Op time: ThuFLEP 106.4 vs. RASP 123.2 min (*p* = 0.012) LOS: ThuFLEP 1.9 vs. RASP 4.9 d (*p* = 0.009) Catheter: ThuFLEP 1.7 vs. RASP 5.0 d (*p* = 0.009) Transfusion: 9 ThuFLEP vs. 1 RASP (raw counts) RASP had significantly larger prostate volumes	Overall: ThuFLEP 12% vs. RASP 2.8% (*p* = 0.022) Clavien ≥ III: ThuFLEP 3.1% vs. RASP 0.94% (*p* = 0.073, NS) Stress UI at 3 months: ThuFLEP 4.7% vs. RASP 1.9% (*p* = 0.2, NS) Caveat: ThuFLEP on learning curve; rates expected to improve	IPSS at 3 months: RASP lower than ThuFLEP (*p* = 0.012) Urgency: lower in RASP; QoL and Qmax: comparable (both NS) Stress UI at 3 months: ThuFLEP 4.7% vs. RASP 1.9% (*p* = 0.2)

**Table 3:** Postoperative Complications Across Included Studies. Abbreviations: AUR = acute urinary retention; BPH = benign prostatic hyperplasia; EBL = estimated blood loss; EAUiaiC = European Association of Urology intraoperative adverse incident classification; Hb = hemoglobin; HoLEP = holmium laser enucleation of the prostate; ICU = intensive care unit; IPSS = International Prostate Symptom Score; LUTS = lower urinary tract symptoms; MI = myocardial infarction; MIS = minimally invasive surgery; NR = not reported; NS = not significant; OSP = open simple prostatectomy; Qmax = maximum urinary flow; RASP = robot-assisted simple prostatectomy; RSP = robotic simple prostatectomy; ThuFLEP = thulium fiber laser enucleation of the prostate; ThuVEP = thulium vapoenucleation; UI = urinary incontinence; UTI = urinary tract infection.

Study	Technique	Overall Complications	High-Grade (Clavien ≥ III)	Bleeding/Transfusion	Urinary Incontinence	Irritative LUTS/Retention	Other Notable Events
Lee et al. 2023 [17]	HoLEP vs. OSP vs. RSP	HoLEP: 9.6% OSP: 9.1% RSP: 23.1% (*p* = 0.1, NS)	HoLEP: 0 Grade ≥ IIIb OSP: 1 Grade IIIb (laparotomy, 16.6%) RSP: 4 Grade IIIb (2 clot evacuation, 2 ureteral stents) No Grade IV/V	Transfusion: HoLEP 1.0% OSP 47.0% RSP 3.1% (*p* < 0.0001) EBL: HoLEP 66 vs. OSP 795 vs. RSP 326 mL (*p* < 0.0001)	Not separately reported per arm IPSS/SHIM data sparse in OSP/RSP	AUR (HoLEP): 4 ED visits Clot retention (HoLEP): 5 readmissions Catheter-assoc. UTI (RASP): 4 readmissions	30-day ED visits: HoLEP 5.7%, OSP 6.1%, RSP 4.6% (NS) Readmission: HoLEP 4.3%, OSP 3.0%, RSP 18.5% (*p* = 0.0002)
Nestler et al. 2019 [20]	ThuVEP vs. RASP vs. OSP	OSP: 42.8% (incl. transfusions) RASP: 25.7% ThuVEP: 2.9%	OSP: 0 Grade ≥ IIIa RASP: 1 Grade IIIa (surgical revision for bleeding) ThuVEP: 1 Grade IIIa (bleeding) No Grade IV/V	Hb drop: OSP 3.0 g/dL RASP 1.5 g/dL ThuVEP 1.2 g/dL Transfusion: OSP 34.4% RASP 9.4% ThuVEP 0%	Early pad use (24 h post-catheter): ThuVEP: median 0 (5/35 needed 1 pad) RASP: median 0 (9/35 needed ≥1) OSP: median 1 (28/35 needed pads) (*p* ≤ 0.001, OSP vs. MIS)	RASP: 2 Grade II (urinary retention after catheter removal) OSP: 1 Grade I (gross hematuria) RASP: 3 Grade I (prolonged hematuria)	OSP: 2 Grade I (wound infection, secondary healing) No revision for wound complications IPSS/QoL improved equally in all arms (*p* > 0.8)
Zhang et al. 2017 [21]	HoLEP vs. RASP	Clavien ≥ III: HoLEP 1.2% RASP 3.1% (*p* = 0.34, NS) Overall rates not fully reported	HoLEP ≥ III: 0.2% MI 0.2% septic shock 0.7% ICU admit (TNN hyponatremia, hypotension) RASP ≥ III: 3.1% small bowel perforation (exploratory lap)	Hb drop: HoLEP 1.8 g/dL RASP 2.5 g/dL (*p* = 0.004) Transfusion: HoLEP 1.8% RASP 9.4% (*p* = 0.03)	Not reported separately (study focused on perioperative outcomes only)	Catheter reinsertion rate reported but not quantified as incontinence/LUTS Long-term voiding outcomes NR	Symptomatic hyponatremia: HoLEP *n* = 2 (0.3%) Septic shock: HoLEP *n* = 1 (0.2%) Small bowel perf.: RASP *n* = 1 (3.1%) No Grade IV/V
Kim et al. 2022 [31]	Transvesical RASP vs. HoLEP	RASP: Grade I–IIIb events in majority HoLEP: Grade I–II Grade IIIb: 1 (RASP) No Grade IV/V	Grade IIIb: RASP: 1 (3.0%) bladder neckcontracture →urethrotomy at 3 monthsHoLEP: 0 Grade IIIb	Hb drop: RASP 1.8 g/dL HoLEP 0.7 g/dL (*p* < 0.01) Transfusion: 0% in both groups Grade I hematuria: RASP *n* = 2, HoLEP *n* = 1	Stress UI 1 mo: RASP 3.0% HoLEP 15.4% (*p* = 0.09, NS) Stress UI 2 mo: RASP 0 HoLEP 15.4% (*p* = 0.03) RASP favored	De novo urgency: RASP 72.7% HoLEP 56.5% (resolved w/meds) De novo urge UI: RASP 36.3% HoLEP 23.0% AUR @ Grade I: HoLEP *n* = 1	Dysuria (Grade II): RASP 0 HoLEP *n* = 3 Clot retention: RASP *n* = 2, HoLEP *n* = 1 Bladder neck contracture (RASP) IPSS/Qmax/QoL: similar (all NS)
Audige et al. 2025 [32]	RASP vs. ThuFLEP	ThuFLEP: 12% RASP: 2.8% (*p* = 0.022) ThuFLEP higher overall rate	Clavien ≥ III: ThuFLEP 3.1% (*n* = 4: 1 secondary morcellation, 2 clot evacuation) RASP 0.94% (*n* = 1: clot evacuation) (*p* = 0.073, NS)	Clavien II: ThuFLEP 8.6% (*n* = 11) RASP 1.9% (*n* = 2) Transfusions: ThuFLEP *n* = 9 (82% of Grade II events) RASP *n* = 1 (50%)	Stress UI 3 mo: ThuFLEP 4.7% RASP 1.9% (*p* = 0.2, NS)	Urgency @ 90d: ThuFLEP higher (*p* = 0.008) No retention rates reported separately	Intraoperative adverse events: ThuFLEP: 3 (Grade 1 EAUiaiC); RASP: 0 No Grade IV/V ThuFLEP on learning curve; rates expected to improve

**Table 4:** Functional Outcomes Across Included Studies. Abbreviations: AUA = American Urological Association; BPH = benign prostatic hyperplasia; F/U = follow-up; HoLEP = holmium laser enucleation of the prostate; IPSS = International Prostate Symptom Score; MIS = minimally invasive surgery; NR = not reported; NS = not significant; OSP = open simple prostatectomy; PSA = prostate-specific antigen; PVR = post-void residual urine volume; Qmax = maximum urinary flow rate; QoL = quality of life; RASP = robot-assisted simple prostatectomy; RSP = robotic simple prostatectomy; SHIM = Sexual Health Inventory for Men; ThuFLEP = thulium fiber laser enucleation of the prostate; ThuVEP = thulium vapoenucleation; UI = urinary incontinence.

Study	Technique	IPSS (Postoperative)	Quality of Life (QoL)	Maximum Flow Rate (Qmax)	Post-Void Residual (PVR)	Postoperative Continence Status
Lee et al. 2023 [17]	HoLEP vs. OSP vs. RSP	NR per arm (IPSS data sparse in OSP/RSP arms;	NR (SHIM scores sparse in OSP/RSP)	NR	NR	Postop PSA: HoLEP 0.69 OSP 1.23 RSP 1.40 ng/mL (*p* = 0.046) UI rates NR per arm; RSP: highest 30 d readmission (18.5%)
Nestler et al. 2019 [20]	ThuVEP vs. RASP vs. OSP	Preop IPSS: All groups ~23 (matched, *p* > 0.2) Postop: significant improvement in all groups (*p* < 0.05) No inter-group difference (*p* > 0.8)	Preop QoL: All groups ~5 (matched, *p* > 0.2) Postop: significant improvement (*p* < 0.05) No difference between groups (*p* > 0.8)	Free flow (Qmax) improved postop in all groups (*p* < 0.05) No significant difference between arms (*p* > 0.8)	Not reported separately	Early pad use (24 h post-catheter): ThuVEP: median 0 (5/35 needed 1 pad; mean 0.12) RASP: median 0 (9/35 needed ≥1; mean 0.46) OSP: median 1 pad (28/35; mean 1.23) OSP vs. MIS: *p* ≤ 0.001 ThuVEP vs. RASP: *p* = 0.53 (NS)
Zhang et al. 2017 [21]	HoLEP vs. RASP	Baseline AUA: HoLEP 20 vs. RASP 24 (NS) Postop IPSS: NR (periop study only)	NR	NR (postop voiding outcomes NR)	NR	Not assessed (periop study only) Catheter reinsertion rate: HoLEP vs. RASP —similar (NS) Authors note: further postop voiding study needed
Kim et al. 2022 [31]	Transvesical RASP vs. HoLEP	Postop IPSS (obstructive score): RASP 9.4 vs. HoLEP 10.9 (*p* = 0.29, NS) Postop IPSS (irritative score): RASP 1.9 vs. HoLEP 3.8 (*p* = 0.11, NS) Both arms: significant improvement vs. preop (*p* < 0.05)	Postop QoL: RASP 2.2 vs. HoLEP 2.0 (*p* = 0.79, NS) Comparable improvement in both groups	Postop Qmax: RASP 13.2 vs. HoLEP 13.4 mL/s (*p* = 0.92, NS) Both groups significantly improved from baseline	Postop PVR: RASP 98.9 vs. HoLEP 127.2 mL (*p* = 0.38, NS) Both groups improved	Stress UI 2 wk: RASP 9.1% HoLEP 15.4% (*p* = 0.09, NS) Stress UI 1 mo: RASP 3.0% HoLEP 15.4% (*p* = 0.09, NS) Stress UI 2 mo: RASP 0% HoLEP 15.4% (*p* = 0.03, sig.) De novo urgency: RASP 72.7% HoLEP 56.5% (resolved w/meds)
Audige et al. 2025 [32]	RASP vs. ThuFLEP	Postop IPSS 3 months: RASP lower than ThuFLEP (*p* = 0.012) Mean decrease from baseline: 6.47 pts both arms (interaction group × time sig.) Both arms: significant improvement (*p* < 0.001)	Postop QoL 3 months: RASP vs. ThuFLEP —comparable (NS) Both arms: significant improvement (*p* < 0.001)	Postop Qmax 3 months: RASP vs. ThuFLEP —comparable (NS) Both groups: significant improvement (*p* < 0.001)	Preop PVR similar between groups (NS) Postop PVR not separately reported at 3-month F/U	Stress UI 3 mo: RASP 1.9% ThuFLEP 4.7% (*p* = 0.2, NS) Urgency 90d: ThuFLEP higher (*p* = 0.008) Both arms safe; RASP numerically better continence

**Table 5:** Catheterization Time Meta-analysis: Leave-one-out.

Study Omitted	k	MD (Days)	95% CI	*t*	*p*	I^2^
Lee et al. 2023 [17]	4	4.77	1.57–7.98	4.74	0.018	98.07%
Nestler et al. 2019 [20]	4	6.70	1.67–11.73	4.24	0.024	98.61%
Zhang et al. 2017 [21]	4	5.62	−0.10–11.34	3.13	0.052	99.26%
Kim et al. 2022 [31]	4	6.07	0.20–11.95	3.29	0.046	99.02%
Audige et al. 2025 [32]	4	6.61	1.40–11.83	4.04	0.027	99.17%

**Table 6:** Postoperative Hospital Length of Stay Meta-analysis: Leave-one-out Method.

Study Omitted	k	MD (Days)	95% CI	t	*p*-Value	I^2^ (%)
Nestler et al. 2019 [20]	4	2.65	0.20–5.11	3.44	0.041	96.3
Audige et al. 2025 [32]	4	2.66	0.21–5.10	3.46	0.041	97.3
Kim et al. 2022 [31]	4	2.27	0.77–3.77	4.80	0.017	91.4
Lee et al. 2023 [17]	4	2.92	0.59–5.25	3.99	0.028	96.5
Zhang et al. 2017 [32]	4	3.14	1.40–4.88	5.73	0.011	95.1

**Table 7:** Publication bias and sensitivity analyses for the pooled perioperative outcomes. Funnel-plot asymmetry was assessed with the Begg–Mazumdar rank correlation and Egger’s regression tests; the Fail-Safe *N* denotes the number of null-result studies required to render the pooled estimate non-significant. Leave-one-out columns summarize the range of pooled mean differences (MD) on sequential omission of each study. Abbreviations: AEEP = anatomic endoscopic enucleation of the prostate; CI = confidence interval; k = number of studies; MD = mean difference; RASP/MISP = robot-assisted/minimally invasive simple prostatectomy.

Outcome (k)	Begg–Mazumdar (r, *p*)	Egger’s Regression (*p*)	Trim-and-Fill	Fail-Safe *N*	Leave-One-Out Range
Catheterization time (k = 5)	r = 0.60, *p* = 0.233	*p* = 0.134	0 studies imputed	Not reported	MD 4.77–6.70 days; significant in 4/5 iterations
Length of stay (k = 5)	r = −0.60, *p* = 0.233	*p* = 0.328	0 studies imputed	1107 (*p* < 0.001)	MD 2.27–3.14 days; significant in all 5 iterations (all *p* ≤ 0.041)
Hemoglobin drop (k = 3)	r = 1.00, *p* = 0.333	t = 8.67, *p* = 0.073	2 studies imputed	30 (*p* < 0.001)	Not performed (k would reduce to 2)

**Table 8:** (**a**) Newcastle–Ottawa Scale checklist for the included comparative cohort studies (maximum 9 stars). ★ = star awarded; — = star not awarded. Selection domain (4 stars): S1 = representativeness of the exposed cohort; S2 = selection of the non-exposed cohort from the same source population; S3 = ascertainment of exposure (surgical/operative records); S4 = demonstration that the outcome was not present at the start of study. Comparability domain (up to 2 stars): C1 = comparability of cohorts on the basis of design or analysis (one star for the principal confounder, a second for additional adjustment, e.g., propensity-score or matched-pair design). Outcome domain (3 stars): O1 = assessment of outcome (record linkage or independent assessment); O2 = follow-up long enough for outcomes to occur; O3 = adequacy of follow-up of cohorts. (**b**). ROBINS-I checklist for the included cohorts. Risk-of-bias judgments: L = Low; M = Moderate; S = Serious. D1 = bias due to confounding; D2 = bias in selection of participants into the study; D3 = bias in classification of interventions; D4 = bias due to deviations from intended interventions; D5 = bias due to missing data; D6 = bias in measurement of outcomes; D7 = bias in selection of the reported result. The overall judgement equals the most severe domain rating; every cohort was rated at moderate overall risk of bias, driven primarily by potential residual confounding (D1) inherent to the non-randomized designs.

(**a**)										
**Study (Year)**	**S1**	**S2**	**S3**	**S4**	**C1**	**O1**	**O2**	**O3**	**Total (/9)**	**Overall**
Zhang et al. 2017 [21]	★	★	★	★	★★	★	—	★	8	Good
Nestler et al. 2019 [20]	★	★	★	★	★★	★	—	—	7	Good
Kim et al. 2022 [31]	★	★	★	★	★	★	★	—	7	Fair
Lee et al. 2023 [17]	★	★	★	—	★★	★	—	—	6	Fair
Audige et al. 2025 [32]	★	★	★	★	★	★	—	—	6	Fair
(**b**)										
**Study (Year)**	**D1**	**D2**	**D3**	**D4**	**D5**	**D6**	**D7**	**Overall**		
Zhang et al. 2017 [21]	M	L	L	L	L	L	L	Moderate		
Nestler et al. 2019 [20]	M	L	L	L	L	L	L	Moderate		
Kim et al. 2022 [31]	M	M	L	L	L	L	L	Moderate		
Lee et al. 2023 [17]	M	L	L	L	M	L	L	Moderate		
Audige et al. 2025 [32]	M	M	L	L	L	M	L	Moderate		

**Table 9:** GRADE (Grading of Recommendations Assessment, Development and Evaluation) evidence profile for the principal pooled outcomes. Because all contributing studies were non-randomized, the certainty of evidence started at low and was assessed across the five downgrading domains (risk of bias, inconsistency, indirectness, imprecision and publication bias); large, consistent and robust effects were considered as upgrading factors. Certainty symbols: ⊕⊕⊕⊕ High; ⊕⊕⊕○ Moderate; ⊕⊕○○ Low; ⊕○○○ Very low. For length of stay the large, dose-consistent effect that remained significant on every leave-one-out iteration offset the inconsistency downgrade, yielding moderate certainty. Abbreviations: AEEP = anatomic endoscopic enucleation of the prostate; CI = confidence interval; I^2^ = inconsistency statistic; MD = mean difference; NS = not statistically significant; RR = risk ratio.

Outcome	No. of Studies (Design)	Risk of Bias	Inconsistency	Indirectness	Imprecision	Publication Bias	Pooled Effect (95% CI)	Certainty
Catheterization time	5 (observational cohorts)	Not serious	Serious (I^2^ = 99.0%; Lee et al. 2023 [17] outlier)	Not serious	Not serious (CI excludes null; *p* = 0.014)	Undetected (Begg *p* = 0.233; Egger *p* = 0.134)	MD 5.95 days (1.98–9.92), favors AEEP	⊕⊕○○ Low
Length of hospital stay	5 (observational cohorts)	Not serious	Serious (I^2^ = 96.1%)	Not serious	Not serious (significant in all leave-one-out)	Undetected (Egger *p* = 0.328; Fail-Safe *N* = 1107)	MD 2.73 days (1.07–4.39), favors AEEP	⊕⊕⊕○ Moderate
Perioperative hemoglobin drop	3 (observational cohorts)	Not serious	Serious (I^2^ = 72.7%)	Not serious	Serious (95% CI crosses null; k = 3)	Suspected (2 studies imputed; Egger *p* = 0.073)	MD 0.65 g/dL (−0.33 to 1.63), NS	⊕⊕○○ Low
Transfusion risk	3 (observational cohorts)	Not serious	Serious (high heterogeneity; sparse events)	Not serious	Very serious (few events; CI crosses null)	Not assessable (k too small)	RR ≈ 0.46 (0.18–1.15), NS	⊕○○○ Very low

## Data Availability

No new data were created or analyzed in this study.

## Supplementary Materials

The following supporting information can be downloaded at: https://www.mdpi.com/article/10.3390/jcm15135276/s1, Table S1: PRISMA checklist.
