# Supplementary material for: Robot-Assisted Simple Prostatectomy Versus Endoscopic Enucleation for Large-Volume Benign Prostatic Hyperplasia: A Systematic Review and Meta-Analysis of Perioperative Outcomes and Complications
Source: J Clin Med. 2026 Jul 6;15(13):5276. doi: 10.3390/jcm15135276 (PMC13362788; doi:10.3390/jcm15135276)
Supplement: Supplementary file 1 [file jcm-15-05276-s001.zip › jcm-4365457-supplementary.pdf]

PRISMA 2020 Checklist

Section and Topic	Item #	Checklist item	Location where item is reported
**TITLE**			
Title	1	Identify the report as a systematic review.	Page 1, Line 2<br>Title identifies the report as: "...A Systematic Review and Meta-Analysis of Perioperative Outcomes and Complications"
**ABSTRACT**			
Abstract	2	See the PRISMA 2020 for Abstracts checklist.	Page 1, Lines 9–35<br>Structured abstract: Background (line 10), Objective (line 14), Methodology (line 17), Results (line 20), Conclusion (line 28).
**INTRODUCTION**			
Rationale	3	Describe the rationale for the review in the context of existing knowledge.	Page 2, Lines 36–59<br>Section 1 – Introduction
Objectives	4	Provide an explicit statement of the objective(s) or question(s) the review addresses.	Page 3, Lines 60–63<br>Section 1.1 – Objective (lines 61–63); Table 1 – PICO Framework (page 3, lines 70–79).
**METHODS**			
Eligibility criteria	5	Specify the inclusion and exclusion criteria for the review and how studies were grouped for the syntheses.	Page 3, Lines 68–80<br>Section 2.2 – Eligibility Criteria (lines 69–79); Table 1 (lines 70–79).
Information sources	6	Specify all databases, registers, websites, organisations, reference lists and other sources searched or consulted to identify studies. Specify the date when each source was last searched or consulted.	Page 3, Lines 80–85<br>Section 2.3 – Search Strategy (lines 81–85).
Search strategy	7	Present the full search strategies for all databases, registers and websites, including any filters and limits used.	Page 3, Lines 81–87<br>Section 2.3 – Search Strategy.
Selection process	8	Specify the methods used to decide whether a study met the inclusion criteria of the review, including how many reviewers screened each record and each report retrieved, whether they worked independently, and if applicable, details of automation tools used in the process.	Page 3–4, Lines 86–89<br>Section 2.4 – Study Selection (lines 87–88).
Data collection process	9	Specify the methods used to collect data from reports, including how many reviewers collected data from each report, whether they worked independently, any processes for obtaining or confirming data from study investigators, and if applicable, details of automation tools used in the process.	Page 4, Lines 89–90<br>Section 2.4 – Data Extraction (lines 89–90).
Data items	10a	List and define all outcomes for which data were sought.	Page 3, Lines 69–79

PRISMA

PRISMA 2020 Checklist

Section and Topic	Item #	Checklist item	Location where item is reported
		Specify whether all results that were compatible with each outcome domain in each study were sought (e.g. for all measures, time points, analyses), and if not, the methods used to decide which results to collect.	Section 2.2 – Eligibility Criteria / Table 1 (lines 70–79).
	10b	List and define all other variables for which data were sought (e.g. participant and intervention characteristics, funding sources). Describe any assumptions made about any missing or unclear information.	Page 4, Lines 89–90<br>Section 2.4 – Data Extraction.
Study risk of bias assessment	11	Specify the methods used to assess risk of bias in the included studies, including details of the tool(s) used, how many reviewers assessed each study and whether they worked independently, and if applicable, details of automation tools used in the process.	Page 4, Lines 91–94<br>Section 2.5 – Risk of Bias Assessment (lines 92–94). Results in Section 3.2 / Table 4 (page 6, line 165).
Effect measures	12	Specify for each outcome the effect measure(s) (e.g. risk ratio, mean difference) used in the synthesis or presentation of results.	Page 4, Lines 95–103<br>Section 2.6 – Statistical Analysis (lines 96–97).
Synthesis methods	13a	Describe the processes used to decide which studies were eligible for each synthesis (e.g. tabulating the study intervention characteristics and comparing against the planned groups for each synthesis (item #5)).	Page 3–4, Lines 69–103<br>Section 2.2 and Section 2.6.
	13b	Describe any methods required to prepare the data for presentation or synthesis, such as handling of missing summary statistics, or data conversions.	Page 4, Lines 96–103<br>Section 2.6 – Statistical Analysis (lines 96–103).
	13c	Describe any methods used to tabulate or visually display results of individual studies and syntheses.	Pages 6–9, Lines 167–241<br>Sections 3.3–3.7; Figures 1a–4a (forest plots) and Tables 2a–2b for individual study data.
	13d	Describe any methods used to synthesize results and provide a rationale for the choice(s). If meta-analysis was performed, describe the model(s), method(s) to identify the presence and extent of statistical heterogeneity, and software package(s) used.	Page 4, Lines 96–103<br>Section 2.6 – Statistical Analysis.
	13e	Describe any methods used to explore possible causes of heterogeneity among study results (e.g. subgroup analysis, meta-regression).	Page 4, Lines 100–103<br>Section 2.6 – Statistical Analysis (lines 100–103).
	13f	Describe any sensitivity analyses conducted to assess robustness of the synthesized results.	Pages 7–9, Lines 179, 200, 218, 237<br>Sections 3.3–3.6; Figures 1c, 2c, 3c–d, 4c.
Reporting bias	14	Describe any methods used to assess risk of bias due to	Pages 6–9, Lines 177, 201, 215, 236

Section and Topic	Item #	Checklist item	Location where item is reported
assessment		missing results in a synthesis (arising from reporting biases).	Sections 3.3–3.6; Figures 1b–4b.
Certainty assessment	15	Describe any methods used to assess certainty (or confidence) in the body of evidence for an outcome.	N/A
**RESULTS**			
Study selection	16a	Describe the results of the search and selection process, from the number of records identified in the search to the number of studies included in the review, ideally using a flow diagram.	Page 4–6, Lines 104–170; Abstract p.1 line 20<br>Section 3.1 – PRISMA flow diagram
	16b	Cite studies that might appear to meet the inclusion criteria, but which were excluded, and explain why they were excluded.	N/A
Study characteristics	17	Cite each included study and present its characteristics.	Pages 4–6, Lines 105–170<br>Section 3.1 / Tables 2a–2b (page 5, lines ~118–141).
Risk of bias in studies	18	Present assessments of risk of bias for each included study.	Pages 5–6, Lines 142–170<br>Section 3.2 – Quality Assessment / Table 4 (page 6, line 165).
Results of individual studies	19	For all outcomes, present, for each study: (a) summary statistics for each group (where appropriate) and (b) an effect estimate and its precision (e.g. confidence/credible interval), ideally using structured tables or plots.	Pages 5–8, Lines 118–237<br>Tables 2a–2b; Figures 1a–4a.
Results of syntheses	20a	For each synthesis, briefly summarise the characteristics and risk of bias among contributing studies.	Pages 6–9, Lines 166–241<br>Sections 3.3–3.7
	20b	Present results of all statistical syntheses conducted. If meta-analysis was done, present for each the summary estimate and its precision (e.g. confidence/credible interval) and measures of statistical heterogeneity. If comparing groups, describe the direction of the effect.	Pages 6–9, Lines 173, 191, 209, 226<br>Sections 3.3–3.6 / Figures 1a–4a.
	20c	Present results of all investigations of possible causes of heterogeneity among study results.	Pages 7–9, Lines 179, 200, 218, 232<br>Sections 3.3–3.6
	20d	Present results of all sensitivity analyses conducted to assess the robustness of the synthesized results.	Pages 7–9, Lines 179, 200, 218, 237<br>Figures 1c, 2c, 3c–d, 4c.
Reporting biases	21	Present assessments of risk of bias due to missing results (arising from reporting biases) for each synthesis assessed.	Pages 6–9, Lines 177, 201, 215, 236<br>Sections 3.3–3.6 / Figures 1b–4b
Certainty of	22	Present assessments of certainty (or confidence) in the	N/A

Section and Topic	Item #	Checklist item	Location where item is reported
evidence		body of evidence for each outcome assessed.	
**DISCUSSION**			
Discussion	23a	Provide a general interpretation of the results in the context of other evidence.	Pages 9–10, Lines 261–297<br>Section 4 – Discussion (lines 261–296).
	23b	Discuss any limitations of the evidence included in the review.	Pages 9–10, Lines 261–296<br>Section 4 – Discussion.
	23c	Discuss any limitations of the review processes used.	Pages 9–10, Lines 261–296<br>Section 4 – Discussion.
	23d	Discuss implications of the results for practice, policy, and future research.	Pages 10–11, Lines 288–297<br>Section 4 – Discussion final para (lines 288–297) / Conclusion (line 297).
**OTHER INFORMATION**			
Registration and protocol	24a	Provide registration information for the review, including register name and registration number, or state that the review was not registered.	N/A
	24b	Indicate where the review protocol can be accessed, or state that a protocol was not prepared.	N/A
	24c	Describe and explain any amendments to information provided at registration or in the protocol.	N/A
Support	25	Describe sources of financial or non-financial support for the review, and the role of the funders or sponsors in the review.	Pages 19
Competing interests	26	Declare any competing interests of review authors.	Pages 19
Availability of data, code and other materials	27	Report which of the following are publicly available and where they can be found: template data collection forms; data extracted from included studies; data used for all analyses; analytic code; any other materials used in the review.	N/A

From: Page MJ, McKenzie JE, Bossuyt PM, Boutron I, Hoffmann TC, Mulrow CD, et al. The PRISMA 2020 statement: an updated guideline for reporting systematic reviews. BMJ 2021;372:n71. doi: 10.1136/bmj.n71. This work is licensed under CC BY 4.0. To view a copy of this license, visit https://creativecommons.org/licenses/by/4.0/
